# Withanolides from *Physalis angulata* L.

**DOI:** 10.1107/S205698902100709X

**Published:** 2021-07-16

**Authors:** R. Ya. Okmanov, M. M. Makhmudova, I. D. Bobaev, B. Tashkhodjaev

**Affiliations:** a S.Yunusov Institute of the Chemistry of Plant Substances Academy of Sciences, of Uzbekistan 100170, Mirzo Ulugbek Str., 77, Tashkent, Uzbekistan; b Tashkent Chemical-Technological Institute, of Uzbekistan 100011, A. Navoiy Str., 32, Tashkent, Uzbekistan

**Keywords:** *Physalis angulata* L., physangulide B, 14α-hy­droxy­ixocarpanolide, mol­ecular structure, hydrogen bonding, chloro­form solvate, crystal structure

## Abstract

The compounds (17*S*,20*R*,22*R*,24*R*,25*S*)-5β,6β:20,24-diep­oxy-4β,25-dihy­droxy-1-oxowith-2-en-26,22-olide and (20*R*,22*R*)-5α,14α,20-Trihy­droxy-1-oxo-6α,7α-ep­oxy­witha-2-enolide were isolated from a chloro­form extract of the aerial parts of *Physalis angulata* L. (Solanaceae). Two products were isolated from the chromatographic separation extract. Compound **I** corresponds to physangulide B chloro­form monosolvate, C_28_H_38_O_7_·CHCl_3_, while compound **II** is 14α-hy­droxy­ixocarpanolide, C_28_H_40_O_7_. In the two mol­ecular structures, the conformation of the steroid part (rings *A*, *B*, *C*, *D*) does not differ. In both crystals, mol­ecules are linked by inter­molecular O—H⋯O hydrogen bonds along the *c-*axis direction and form a two-dimensional network parallel to the *ac* plane. The absolute configuration was determined from X-ray diffraction data.

## Abstract 2

The chemical structures of two withanolides, isolated from the leaves of *Physalis angulata* by column chromatography, were studied. The isolated compounds are (17*S*,20*R*,22*R*,24*R*,25*S*)-5β,6β:20,24-diep­oxy-4β,25-dihy­droxy-1-oxowith-2-en-26,22-olide and (20*R*,22*R*)-5α,14α,20-Trihy­droxy-1-oxo-6α,7α-ep­oxy­witha-2-enolide.

## Chemical context   

The genus *Physalis* belongs to the nightshade family of plants and is widely distributed in subtropical and tropical regions around the world. Some *Physalis* species are important in the diet because of their edible fruits. Phytochemical and pharmacological studies show that in plants of the genus *Physalis*, the main biological substances are withanolides (Huang *et al.*, 2020 ▸). The fruits of *Physalis angulata* L. are edible, traditionally collected from wild populations, but the plant is now widely cultivated. In different countries of the world the fruits, roots and leaves of *Physalis angulata* L. are used in folk medicine as a treatment for various diseases (Salgado & Arana, 2013 ▸). The main secondary metabolites of *Physalis angulata* are withanolides, which are highly variable in chemical structure and exhibit inter­esting pharmacological activity (Ray & Gupta, 1994 ▸; Figueiredo *et al.*, 2020 ▸; Sá *et al.*, 2011 ▸; Pinto *et al.*, 2016 ▸). The plant *Physalis angulata* is widespread in Uzbekistan and its reserves are sufficient for industrial use (Vasina *et al.*, 1990 ▸). 
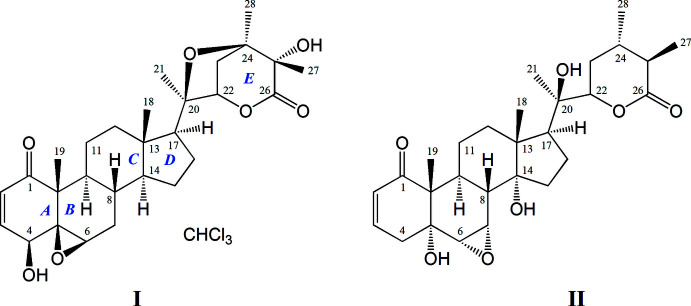


To study the chemical structure of withanolides, leaves of *Physalis angulata* collected in the Tashkent region were used. Isolation of withanolides from the leaves of *Physalis angulata* and separation of components into individual substances was carried out by column chromatography. The isolated compounds were identified as physangulide B chloro­form solvate (**I**) and 14α-hy­droxy­ixocarpanolide (**II**).

## Structural commentary   

**Figure 1:**
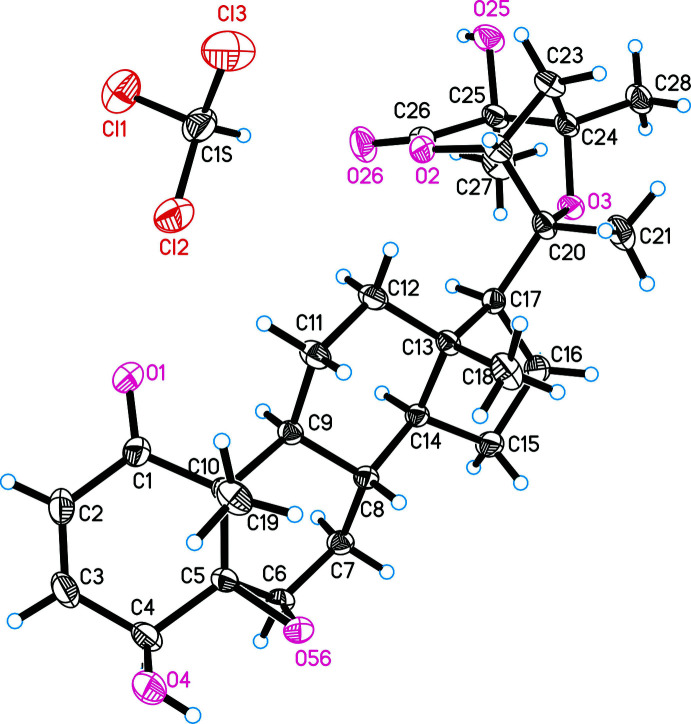
Mol­ecular structure of the chloro­form solvate of physangulide B (compound **I**), including atom labelling. Displacement ellipsoids are drawn at the 30% probability level.

The asymmetric unit of **I**, a chloro­form solvate of physangulide B, is shown in Fig. 1 ▸. The use of Cu *K*α radiation allowed the determination of the absolute configuration of the physangulide B mol­ecule. The Flack parameter refined to 0.014 (6). The chiral centres of the physangulide B mol­ecule have the following chirality: 4*S*, 5*R*, 6*R*, 8*S*, 9*S*,10*R*, 13*S*, 14*S*, 17*S*, 20*R*, 22*R*, 24*R* and 25*S*. The stereochemistry of physangulide B [systematic name (17*S*,20*R*,22*R*,24*R*,25*S*)-5β,6β:20,24-diep­oxy-4β,25-dihy­droxy-1-oxowith-2-en-26,22-olide] does not differ from that found for the acetyl derivative and confirms the absolute configuration proposed earlier for physangulide B (Maldonado *et al.*, 2015 ▸).

**Figure 2:**
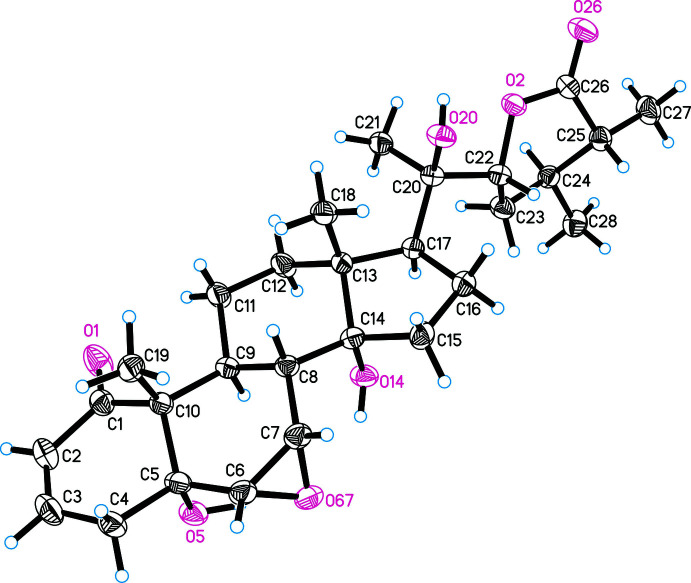
Mol­ecular structure of the 14α-hy­droxy­ixocarpanolide (compound **II**), including atom labelling. Displacement ellipsoids are drawn at the 30% probability level.

The mol­ecular structure of withanolide **II** is shown in Fig. 2 ▸. The Flack parameter refined to −0.1 (2) and allowed the absolute configuration of **II** to be confirmed. The chiral centres in the mol­ecule have the following chirality: 5*R*, 6*S*, 7*S*, 8*S*, 9*S*, 10*R*, 13*R*, 14*R*, 17*S*, 20*R*, 22*R*, 24*S*, 25*R*. According to the experimental data, the isolated compound is 14α-hy­droxy­ixocarpanolide [systematic name: (20*R*,22*R*)-5α,14α,20-trihy­droxy-1-oxo-6α,7α-ep­oxy­witha-2-enolide (Vasina *et al.*, 1986 ▸; Ray & Gupta, 1994 ▸).

In both mol­ecules, ring *C* adopts a chair conformation and ring *D* an envelope conformation with atom C13 as the flap. Ring *A* exhibits a half–chair conformation, but differs slightly in the arrangement of atoms. The C1–C4 fragment is planar with r.m.s deviations of 0.0045 Å for **I** and 0.034 Å for **II**. The deviations of atoms C5 and C10 atoms from this plane are −0.225 (7) and 0.291 (7) Å, respectively, for **I** and −0.478 (7) and 0.280 (7) Å for **II**.

In the mol­ecule of **I**, atoms of ring *B* are located in one plane (with an r.m.s deviation of 0.0132 Å), except for C8 which deviates from the plane of the remaining atoms by 0.666 (4) Å. A similar envelope conformation for ring *B* is observed in **II**. Here, the C5–C9 atoms are located in one plane with an accuracy of 0.0643 Å, atom C10 being displaced from the plane through the remaining atoms by 0.696 (4) Å. This difference in the arrangement of atoms in planes is explained by the position of the ep­oxy bridge, which is located in the β-position for **I** and the α-position for **II**.

## Supra­molecular features   

**Figure 3:**
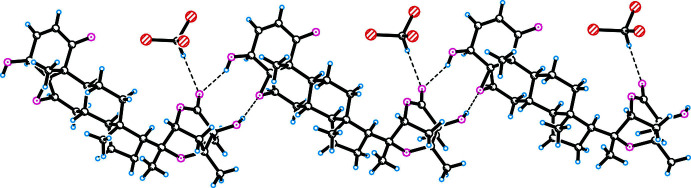
Hydrogen bonding in the crystal structure of **I** (the mol­ecules are cross-linked along the *c* axis).

In both crystal structures, inter­molecular hydrogen bonds of the O—H⋯O type are observed, which link the mol­ecules along the *c-*axis direction. In compound **I**, O—H⋯O and C—H⋯O hydrogen bonds are observed between mol­ecules of physangulide B (Table 1 ▸). O4—H4⋯O26 and O25—H25⋯O56 hydrogen bonds are involved in the formation of an infinite chain along the *c*-axis (Fig. 3 ▸). In addition, the chloro­form mol­ecule participates in a hydrogen bond with the oxygen atom O26 of the lactone fragment. A similar hydrogen bond with a solvate mol­ecule (methanol) is observed in the structure of the acetyl derivative of physangulide B (FUQKAF; Maldonado *et al.*, 2015 ▸).

**Figure 4:**
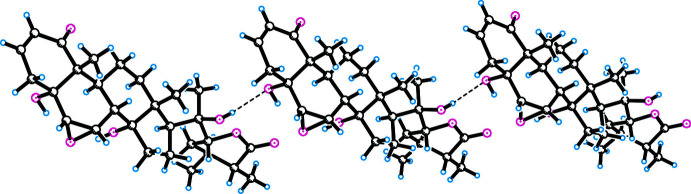
O20—H20⋯O5 hydrogen bonds in the crystal structure of compound **II** (the mol­ecules are cross-linked along the *c* axis).

Similar inter­molecular O—H⋯O and C—H⋯O hydrogen bonds are observed in the structure of **II** (Table 2 ▸). The formation of an infinite O20—H20⋯O5 hydrogen-bonded chain is shown in Fig. 4 ▸. Paired hydrogen bonds are observed between mol­ecules, which extend along the *c-*axis direction.

## Database survey   

A search for related structures (*A*, *B*, *C*, *D*, *E* rings are connected according to the order of the studied compounds) in the Cambridge Structural Database (CSD Version 5.42, update of November 2020; Groom *et al.*, 2016 ▸) resulted in eleven hits. Of the structures found, the closest structure considering the connectivity and chirality of atoms is 17-(4-hy­droxy-4,5,7-trimethyl-3-oxo-2,6-dioxabi­cyclo­[3.2.1]oct-7-yl)-1-oxo-5,6-ep­oxy­androst-2-en-4-yl acetate methanol solvate (FUQKAF; Maldonado *et al.*, 2015 ▸). Structures with the 5β,6β-ep­oxy-4β-hy­droxy groups in the same location in the mol­ecule as the title compounds are 4,16,20,24-tetra­hydroxy-5,6:22,26-di­epoxy­ergost-2-ene-1,26-dione methanol solvate (GANFOS, Maldonado *et al.*, 2011 ▸), (17*R*,20*R*,22*R*,24*S*,25*R*)-4β,17α,20β-trihy­droxy-5β,6β-ep­oxy-1-oxowitha-2-en-26,22-olide (Philadelphicalactone A, XIVYEG; Su *et al.*, 2002 ▸) and (17*R*,20*S*,22*R*)-4β-hy­droxy-1-oxo-5β,6β-ep­oxy-16α-acet­oxy­witha-2-enolide ethyl acetate clathrate (YISSOI; Alfonso *et al.*, 1993 ▸).

## Synthesis and crystallization   

**Isolation of individual substances from the leaves of** ***Physalis angulata***

Collected dried leaves (4 kg) of *Physalis angulata* L. were poured into cold water and heated to boiling. The hot mass was squeezed out through a canvas. The plant was again poured into cold water, heated, and the hot mass was squeezed out through the canvas again. The water extract was distilled until the volume decreased to 3 L. Chloro­form (3 L) was poured into the received solution and substances were extracted. From the chloro­form layer, insoluble and soluble substances (25 g) were isolated. To the isolated dry mass, 0.5 L of chloro­form were added and the solution was filtered (the mass of the insoluble compounds was 5.8 g). From the filtrate after distillation, 19.2 g of compounds were isolated. The compounds isolated from the filtrate were loaded onto a column containing 0.5 kg of silica gel (Silica gel 60, 0.063-0.1 mm, Merck). The sums of substances were eluted with system 1 (chloro­form:methanol 99:1) to produce fractions 1–5, and eluted with system 2 (chloro­form:methanol 97:3) to produce fractions 5–9. The process was monitored by thin layer chromatography (Silica gel on TLC Al foils, fluorescent indicator 254 nm). Fractions 2–4 (6.8 g) and 6–8 (4.0 g) were shown by TLC to consist of individual substances.

The obtained fractions were purified by repeated chromatography. Rechromatography of fractions 2–4 containing physangulide B in system 3 (chloro­form:methanol 10:1) gave 5.96 g of the individual product. The *R*_F_ in system 3 was 0.58, visualized as a crimson spot. The yield was 0.15%, based on the weight of the air-dry raw material. Rechromatography of fractions 6–8 in system 1 yielded 3.6 g of 14α-hy­droxy­ixocarpanolide, *R*_F_ = 0.34 in system 2, visualized as a pink spot. The yield was 0.028%.

**Physangulide B [(17** ***S*** **,20** ***R*** **,22** ***R*** **,24** ***R*** **,25** ***S*** **)-5β,6β:20,24-diep­oxy-4β,25-dihy­droxy-1-oxowith-2-en-26,22-olide]**

C_28_H_40_O_7_ (methanol), m.p. 553–555 K, [α]^20^_D_ = −56.0 (*c* = 0.21, CHCl_3_). UV spectrum, λ_CH3OHmax_ (logɛ 5600) 212 (4.00) nm. IR (FT–IR, νKBr_max_, cm^−1^): 3411 (*v br*, O—H *str*), 2958 (*m*, C_sycl_—H *str*), 1706 (*s*, C=O *str*), 1676 (*v s*, C=O), 1457 (*m*), 1380 (*m*), 1272 (*s*), 1101 (*s*), 1085 (*m*), 1024 (*m*), 962 (*m*), 921 (*w*), 905 (*w*).

**14α-hy­droxy­ixocarpanolide (5α,14α,20*R*-trihy­droxy-1-oxo-6α,7α-ep­oxywitha-2-enolide)**

C_28_H_40_O_7_ (methanol), mp. 528–530 K, [α]^20^_D_ = + 29.1 ±2 (*c* = 1.18, CHCl_3_). UV spectrum, λ_C2H5OHmax_: 225 nm (ɛ 10370). IR (FT–IR, νKBr_max_, cm^−1^): 3584–3477 (*v br*, O—H *str*), 2949 (*m*, C_sycl_—H *str*), 1754 (*v s*, C=O *str*), 1458 (*w*), 1388 (*m*), 1261 (*s*), 1182 (*m*), 1094 (*v*), 1034 (*m*), 910 (*m*).

## Refinement   

Crystal data, data collection and structure refinement details are summarized in Table 3 ▸. The H atoms bonded to C atoms were placed geometrically (with C—H distances of 0.98 Å for CH, 0.97 Å for CH_2_, 0.96 Å for CH_3_ and 0.93 Å for C_ar_) and included in the refinement in a riding-motion approximation with *U*_iso_(H) = 1.2*U*_eq_(C) [*U*_iso_ = 1.5*U*_eq_(C) for methyl H atoms]. The hydrogen atoms on the O atoms were located in difference-Fourier maps and refined freely.

## Supplementary Material

Crystal structure: contains datablock(s) I, II, Global. DOI: 10.1107/S205698902100709X/dj2029sup1.cif

Structure factors: contains datablock(s) I. DOI: 10.1107/S205698902100709X/dj2029Isup2.hkl

Structure factors: contains datablock(s) II. DOI: 10.1107/S205698902100709X/dj2029IIsup3.hkl

CCDC references: 2095478, 2095477

Additional supporting information:  crystallographic information; 3D view; checkCIF report

## Figures and Tables

**Table 1:** Hydrogen-bond geometry (Å, °) for (I)

*D*—H⋯*A*	*D*—H	H⋯*A*	*D*⋯*A*	*D*—H⋯*A*
O4—H4⋯O26^i^	0.79 (6)	2.10 (6)	2.819 (4)	151
O25—H25⋯O56^ii^	0.74 (5)	2.12 (5)	2.856 (4)	169
C23—H23*A*⋯O26^iii^	0.97	2.57	3.473 (4)	154
C1*S*—H1*SA*⋯O26	0.98	2.43	3.393 (6)	168

Symmetry codes: (i) x, y, z+1; (ii) x, y, z-1; (iii) x+1, y, z.

**Table 2:** Hydrogen-bond geometry (Å, °) for (II)

*D*—H⋯*A*	*D*—H	H⋯*A*	*D*⋯*A*	*D*—H⋯*A*
O20—H20⋯O5^i^	0.76 (6)	2.22 (6)	2.973 (4)	173
C7—H7*A*⋯O26^ii^	0.98	2.59	3.367 (5)	136

Symmetry codes: (i) x, y, z+1; (ii) x-1, y, z-1.

**Table 3:** Experimental details

	(I)	(II)
Crystal data		
Chemical formula	C_28_H_38_O_7_·CHCl_3_	C_28_H_40_O_7_
*M*_r_	605.95	488.60
Crystal system, space group	Monoclinic, *P*2_1_	Triclinic, *P*1
Temperature (K)	290	290
*a*, *b*, *c* (Å)	7.3633 (15), 15.952 (3), 12.657 (3)	6.2374 (12), 9.5938 (19), 11.351 (2)
α, β, γ (°)	90, 94.14 (3), 90	112.81 (3), 96.49 (3), 93.13 (3)
*V* (Å^3^)	1482.9 (5)	618.5 (2)
*Z*	2	1
Radiation type	Cu *K*α	Cu *K*α
μ (mm^−1^)	3.17	0.76
Crystal size (mm)	0.50 × 0.34 × 0.31	0.42 × 0.28 × 0.21

Data collection		
Diffractometer	Rigaku Xcalibur, Ruby	Rigaku Xcalibur, Ruby
Absorption correction	Multi-scan (*SADABS*; Sheldrick, 2003 ▸)	Multi-scan (*SADABS*; Sheldrick, 2003 ▸)
*T*_min_, *T*_max_	0.316, 0.376	0.776, 0.853
No. of measured, independent and observed [*I* > 2σ(*I*)] reflections	13820, 5743, 5622	4169, 2812, 2545
*R*_int_	0.028	0.025
(sin θ/λ)_max_ (Å^−1^)	0.629	0.631

Refinement		
*R*[*F* ^2^ > 2σ(*F* ^2^)], *wR*(*F* ^2^), *S*	0.042, 0.113, 1.04	0.041, 0.110, 1.04
No. of reflections	5743	2812
No. of parameters	365	333
No. of restraints	1	3
H-atom treatment	H atoms treated by a mixture of independent and constrained refinement	H atoms treated by a mixture of independent and constrained refinement
Δρ_max_, Δρ_min_ (e Å^−3^)	0.41, −0.34	0.17, −0.19
Absolute structure	Flack *x* determined using 2401 quotients [(*I* ^+^)−(*I* ^−^)]/[(*I* ^+^)+(*I* ^−^)] (Parsons *et al.*, 2013 ▸)	Classical Flack method preferred over Parsons because s.u. lower
Absolute structure parameter	0.014 (6)	−0.1 (2)

Computer programs: *CrysAlis PRO* (Rigaku OD, 2018 ▸), *SHELXS7* (Sheldrick, 2008 ▸), *SHELXL2014/8* (Sheldrick, 2015 ▸), *XP* (Sheldrick, 1998 ▸), *SHELXTL* (Sheldrick, 2008 ▸), *PLATON* (Spek, 2020 ▸) and *publCIF* (Westrip, 2010 ▸)’.

## supplementary crystallographic information

### (17S,20R,22R,24R,25S)-5β,6β:20,24-Diepoxy-4β,25-dihydroxy-1-oxowith-2-en-26,22-olide chloroform solvate (I) Crystal data

**Table:**

C_28_H_38_O_7_·CHCl_3_	*D*_x_ = 1.357 Mg m^−^^3^
*M**_r_* = 605.95	Melting point: 553(2) K
Monoclinic, *P*2_1_	Cu *K*α radiation, λ = 1.54184 Å
*a* = 7.3633 (15) Å	Cell parameters from 7825 reflections
*b* = 15.952 (3) Å	θ = 5.5–76.0°
*c* = 12.657 (3) Å	µ = 3.17 mm^−^^1^
β = 94.14 (3)°	*T* = 290 K
*V* = 1482.9 (5) Å^3^	Prizmatic, colorless
*Z* = 2	0.50 × 0.34 × 0.31 mm
*F*(000) = 640	

### (17S,20R,22R,24R,25S)-5β,6β:20,24-Diepoxy-4β,25-dihydroxy-1-oxowith-2-en-26,22-olide chloroform solvate (I) Data collection

**Table:**

Rigaku Xcalibur, Ruby diffractometer	5743 independent reflections
Radiation source: Enhance (Cu) X-ray Source	5622 reflections with *I* > 2σ(*I*)
Graphite monochromator	*R*_int_ = 0.028
Detector resolution: 10.2576 pixels mm^-1^	θ_max_ = 75.9°, θ_min_ = 3.5°
ω scans	*h* = −9→9
Absorption correction: multi-scan (SADABS; Sheldrick, 2003)	*k* = −19→19
*T*_min_ = 0.316, *T*_max_ = 0.376	*l* = −13→15
13820 measured reflections	

### (17S,20R,22R,24R,25S)-5β,6β:20,24-Diepoxy-4β,25-dihydroxy-1-oxowith-2-en-26,22-olide chloroform solvate (I) Refinement

**Table:**

Refinement on *F*^2^	Hydrogen site location: mixed
Least-squares matrix: full	H atoms treated by a mixture of independent and constrained refinement
*R*[*F*^2^ > 2σ(*F*^2^)] = 0.042	*w* = 1/[σ^2^(*F*_o_^2^) + (0.0643*P*)^2^ + 0.4392*P*] where *P* = (*F*_o_^2^ + 2*F*_c_^2^)/3
*wR*(*F*^2^) = 0.113	(Δ/σ)_max_ < 0.001
*S* = 1.04	Δρ_max_ = 0.41 e Å^−^^3^
5743 reflections	Δρ_min_ = −0.34 e Å^−^^3^
365 parameters	Absolute structure: Flack *x* determined using 2401 quotients [(*I*^+^)-(*I*^-^)]/[(*I*^+^)+(*I*^-^)] (Parsons *et al.*, 2013)
1 restraint	Absolute structure parameter: 0.014 (6)
Primary atom site location: structure-invariant direct methods	

### (17S,20R,22R,24R,25S)-5β,6β:20,24-Diepoxy-4β,25-dihydroxy-1-oxowith-2-en-26,22-olide chloroform solvate (I) Special details

**Table:**

Geometry. All esds (except the esd in the dihedral angle between two l.s. planes) are estimated using the full covariance matrix. The cell esds are taken into account individually in the estimation of esds in distances, angles and torsion angles; correlations between esds in cell parameters are only used when they are defined by crystal symmetry. An approximate (isotropic) treatment of cell esds is used for estimating esds involving l.s. planes.

### (17S,20R,22R,24R,25S)-5β,6β:20,24-Diepoxy-4β,25-dihydroxy-1-oxowith-2-en-26,22-olide chloroform solvate (I) Fractional atomic coordinates and isotropic or equivalent isotropic displacement parameters (Å^2^)

**Table:**

	*x*	*y*	*z*	*U*_iso_*/*U*_eq_	
O1	0.5252 (5)	0.9171 (2)	0.5852 (2)	0.0678 (9)	
O2	1.0029 (3)	0.76628 (14)	0.21727 (16)	0.0380 (5)	
O3	1.1887 (3)	0.60670 (14)	0.22825 (16)	0.0357 (4)	
O4	0.5431 (5)	0.8210 (2)	0.9630 (2)	0.0619 (8)	
O25	1.0440 (4)	0.6971 (2)	−0.02618 (18)	0.0534 (7)	
O26	0.7584 (3)	0.7338 (2)	0.1201 (2)	0.0598 (7)	
O56	0.7082 (3)	0.70353 (16)	0.84614 (16)	0.0419 (5)	
C1	0.5220 (5)	0.8852 (2)	0.6718 (3)	0.0402 (7)	
C2	0.3764 (5)	0.9089 (3)	0.7396 (3)	0.0530 (9)	
H2A	0.3045	0.9551	0.7202	0.064*	
C3	0.3425 (5)	0.8679 (3)	0.8265 (3)	0.0513 (9)	
H3A	0.2463	0.8858	0.8646	0.062*	
C4	0.4509 (5)	0.7944 (2)	0.8665 (3)	0.0442 (7)	
H4B	0.3664	0.7494	0.8824	0.053*	
C5	0.5835 (4)	0.7603 (2)	0.7888 (2)	0.0322 (6)	
C6	0.5780 (4)	0.6705 (2)	0.7655 (2)	0.0352 (6)	
H6A	0.4750	0.6394	0.7912	0.042*	
C7	0.6634 (4)	0.63329 (18)	0.6726 (2)	0.0330 (6)	
H7A	0.5713	0.6264	0.6145	0.040*	
H7B	0.7116	0.5783	0.6915	0.040*	
C8	0.8166 (4)	0.68855 (17)	0.63666 (19)	0.0271 (5)	
H8A	0.9182	0.6886	0.6911	0.032*	
C9	0.7496 (4)	0.77894 (17)	0.6166 (2)	0.0277 (5)	
H9A	0.6484	0.7747	0.5622	0.033*	
C10	0.6687 (4)	0.82253 (18)	0.7140 (2)	0.0298 (5)	
C11	0.8951 (5)	0.83293 (19)	0.5672 (3)	0.0394 (7)	
H11A	0.8440	0.8877	0.5499	0.047*	
H11B	0.9972	0.8408	0.6191	0.047*	
C12	0.9643 (5)	0.79385 (19)	0.4669 (3)	0.0385 (7)	
H12A	0.8658	0.7922	0.4117	0.046*	
H12B	1.0607	0.8285	0.4420	0.046*	
C13	1.0362 (3)	0.70547 (18)	0.4875 (2)	0.0283 (5)	
C14	0.8816 (4)	0.65438 (16)	0.53265 (19)	0.0269 (5)	
H14A	0.7772	0.6588	0.4804	0.032*	
C15	0.9460 (5)	0.5629 (2)	0.5285 (3)	0.0420 (7)	
H15A	0.8432	0.5248	0.5209	0.050*	
H15B	1.0208	0.5483	0.5921	0.050*	
C16	1.0582 (5)	0.5598 (2)	0.4299 (3)	0.0401 (7)	
H16A	1.0015	0.5225	0.3767	0.048*	
H16B	1.1809	0.5402	0.4490	0.048*	
C17	1.0614 (4)	0.65077 (18)	0.3875 (2)	0.0292 (5)	
H17A	0.9476	0.6571	0.3431	0.035*	
C18	1.2062 (4)	0.7062 (3)	0.5657 (3)	0.0479 (8)	
H18A	1.1720	0.7221	0.6347	0.072*	
H18B	1.2929	0.7458	0.5422	0.072*	
H18C	1.2596	0.6513	0.5691	0.072*	
C19	0.8116 (5)	0.8745 (2)	0.7823 (3)	0.0434 (7)	
H19A	0.7590	0.8937	0.8451	0.065*	
H19B	0.8486	0.9219	0.7423	0.065*	
H19C	0.9157	0.8401	0.8016	0.065*	
C20	1.2141 (4)	0.6683 (2)	0.3131 (2)	0.0332 (6)	
C21	1.4095 (4)	0.6596 (3)	0.3637 (3)	0.0509 (9)	
H21A	1.4926	0.6545	0.3090	0.076*	
H21B	1.4176	0.6105	0.4078	0.076*	
H21C	1.4401	0.7082	0.4059	0.076*	
C22	1.1939 (4)	0.7507 (2)	0.2501 (2)	0.0371 (6)	
H22A	1.2487	0.7980	0.2902	0.045*	
C23	1.2941 (4)	0.7319 (2)	0.1529 (3)	0.0406 (7)	
H23A	1.4249	0.7303	0.1687	0.049*	
H23B	1.2645	0.7717	0.0964	0.049*	
C24	1.2164 (4)	0.6451 (2)	0.1265 (2)	0.0336 (6)	
C25	1.0239 (4)	0.6579 (2)	0.0736 (2)	0.0378 (6)	
C26	0.9179 (4)	0.7211 (2)	0.1396 (2)	0.0389 (7)	
C27	0.9140 (6)	0.5779 (3)	0.0607 (3)	0.0568 (9)	
H27A	0.9777	0.5384	0.0197	0.085*	
H27B	0.7975	0.5900	0.0251	0.085*	
H27C	0.8972	0.5545	0.1292	0.085*	
C28	1.3367 (5)	0.5895 (3)	0.0646 (3)	0.0470 (8)	
H28A	1.4541	0.5844	0.1021	0.070*	
H28B	1.3501	0.6138	−0.0038	0.070*	
H28C	1.2822	0.5351	0.0560	0.070*	
C1S	0.6099 (7)	0.9209 (3)	0.2081 (4)	0.0664 (11)	
H1SA	0.6333	0.8640	0.1832	0.080*	
Cl1	0.4179 (2)	0.96088 (8)	0.13542 (10)	0.0782 (4)	
Cl2	0.5718 (2)	0.91662 (10)	0.34355 (10)	0.0863 (4)	
Cl3	0.8008 (3)	0.98341 (14)	0.18974 (14)	0.1059 (5)	
H4	0.605 (7)	0.785 (4)	0.989 (4)	0.063 (15)*	
H25	0.958 (7)	0.692 (3)	−0.060 (4)	0.051 (13)*	

### (17S,20R,22R,24R,25S)-5β,6β:20,24-Diepoxy-4β,25-dihydroxy-1-oxowith-2-en-26,22-olide chloroform solvate (I) Atomic displacement parameters (Å^2^)

**Table:**

	*U*^11^	*U*^22^	*U*^33^	*U*^12^	*U*^13^	*U*^23^
O1	0.085 (2)	0.0663 (18)	0.0546 (15)	0.0371 (16)	0.0221 (14)	0.0254 (14)
O2	0.0421 (11)	0.0384 (11)	0.0345 (10)	0.0095 (9)	0.0089 (8)	0.0018 (9)
O3	0.0427 (11)	0.0366 (11)	0.0287 (9)	0.0043 (9)	0.0091 (8)	−0.0020 (8)
O4	0.080 (2)	0.075 (2)	0.0318 (12)	0.0277 (17)	0.0112 (12)	−0.0065 (12)
O25	0.0432 (13)	0.088 (2)	0.0295 (11)	0.0046 (13)	0.0045 (10)	0.0098 (12)
O26	0.0373 (12)	0.091 (2)	0.0506 (14)	0.0193 (13)	0.0029 (10)	−0.0003 (14)
O56	0.0490 (12)	0.0505 (13)	0.0262 (9)	0.0114 (10)	0.0030 (8)	0.0049 (9)
C1	0.0474 (17)	0.0348 (15)	0.0387 (15)	0.0085 (13)	0.0053 (13)	0.0011 (12)
C2	0.0483 (19)	0.059 (2)	0.053 (2)	0.0247 (17)	0.0064 (15)	0.0003 (17)
C3	0.0397 (17)	0.069 (2)	0.0473 (18)	0.0138 (17)	0.0137 (14)	−0.0081 (16)
C4	0.0468 (17)	0.0505 (19)	0.0378 (16)	0.0021 (15)	0.0187 (13)	−0.0022 (13)
C5	0.0333 (13)	0.0391 (15)	0.0246 (12)	0.0024 (12)	0.0054 (10)	0.0014 (11)
C6	0.0389 (14)	0.0351 (14)	0.0329 (13)	−0.0017 (12)	0.0103 (11)	0.0062 (11)
C7	0.0392 (15)	0.0277 (13)	0.0328 (13)	−0.0013 (11)	0.0073 (11)	0.0025 (10)
C8	0.0294 (12)	0.0292 (13)	0.0227 (11)	0.0003 (10)	0.0024 (9)	0.0000 (9)
C9	0.0314 (13)	0.0270 (13)	0.0250 (12)	−0.0003 (10)	0.0047 (9)	−0.0021 (9)
C10	0.0322 (13)	0.0309 (13)	0.0273 (12)	0.0023 (11)	0.0081 (10)	−0.0029 (10)
C11	0.0497 (18)	0.0273 (14)	0.0438 (16)	−0.0060 (13)	0.0213 (13)	−0.0064 (12)
C12	0.0487 (17)	0.0290 (14)	0.0405 (16)	−0.0010 (12)	0.0211 (13)	0.0006 (11)
C13	0.0265 (12)	0.0343 (14)	0.0244 (11)	0.0002 (10)	0.0040 (9)	−0.0038 (10)
C14	0.0303 (12)	0.0259 (13)	0.0246 (11)	0.0012 (10)	0.0030 (9)	0.0002 (9)
C15	0.059 (2)	0.0291 (15)	0.0398 (15)	0.0091 (14)	0.0154 (14)	0.0044 (12)
C16	0.0487 (17)	0.0335 (15)	0.0393 (15)	0.0095 (13)	0.0118 (13)	−0.0007 (12)
C17	0.0288 (12)	0.0326 (14)	0.0260 (12)	0.0038 (10)	0.0008 (9)	−0.0025 (10)
C18	0.0309 (14)	0.075 (2)	0.0369 (15)	−0.0029 (16)	−0.0012 (11)	−0.0169 (16)
C19	0.0470 (17)	0.0451 (17)	0.0389 (15)	−0.0091 (14)	0.0085 (13)	−0.0156 (13)
C20	0.0280 (12)	0.0431 (16)	0.0285 (12)	0.0027 (11)	0.0028 (10)	−0.0063 (11)
C21	0.0295 (14)	0.083 (3)	0.0400 (16)	0.0072 (16)	0.0036 (12)	−0.0052 (17)
C22	0.0344 (14)	0.0407 (16)	0.0369 (14)	−0.0052 (12)	0.0073 (11)	−0.0061 (12)
C23	0.0353 (15)	0.0499 (18)	0.0379 (15)	−0.0082 (13)	0.0112 (12)	−0.0015 (13)
C24	0.0305 (13)	0.0444 (16)	0.0267 (12)	0.0014 (12)	0.0067 (10)	−0.0012 (11)
C25	0.0340 (14)	0.0517 (18)	0.0284 (13)	−0.0010 (13)	0.0072 (10)	0.0006 (12)
C26	0.0361 (15)	0.0492 (18)	0.0322 (14)	0.0049 (13)	0.0071 (11)	0.0087 (12)
C27	0.048 (2)	0.066 (2)	0.057 (2)	−0.0129 (18)	0.0001 (16)	−0.0146 (19)
C28	0.0440 (17)	0.060 (2)	0.0383 (16)	0.0092 (15)	0.0133 (13)	−0.0056 (14)
C1S	0.092 (3)	0.048 (2)	0.060 (2)	0.014 (2)	0.009 (2)	0.0038 (19)
Cl1	0.1089 (10)	0.0598 (6)	0.0632 (6)	0.0123 (6)	−0.0128 (6)	−0.0165 (5)
Cl2	0.1101 (10)	0.0872 (9)	0.0626 (6)	0.0276 (8)	0.0136 (6)	0.0239 (6)
Cl3	0.1052 (11)	0.1223 (14)	0.0904 (10)	−0.0186 (10)	0.0085 (8)	0.0238 (9)

### (17S,20R,22R,24R,25S)-5β,6β:20,24-Diepoxy-4β,25-dihydroxy-1-oxowith-2-en-26,22-olide chloroform solvate (I) Geometric parameters (Å, º)

**Table:**

O1—C1	1.211 (4)	C13—C17	1.559 (4)
O2—C26	1.338 (4)	C14—C15	1.536 (4)
O2—C22	1.459 (4)	C14—H14A	0.9800
O3—C24	1.454 (3)	C15—C16	1.547 (4)
O3—C20	1.458 (3)	C15—H15A	0.9700
O4—C4	1.418 (5)	C15—H15B	0.9700
O4—H4	0.79 (6)	C16—C17	1.547 (4)
O25—C25	1.427 (4)	C16—H16A	0.9700
O25—H25	0.74 (5)	C16—H16B	0.9700
O26—C26	1.200 (4)	C17—C20	1.544 (4)
O56—C5	1.447 (4)	C17—H17A	0.9800
O56—C6	1.448 (4)	C18—H18A	0.9600
C1—C2	1.470 (5)	C18—H18B	0.9600
C1—C10	1.539 (4)	C18—H18C	0.9600
C2—C3	1.319 (6)	C19—H19A	0.9600
C2—H2A	0.9300	C19—H19B	0.9600
C3—C4	1.486 (5)	C19—H19C	0.9600
C3—H3A	0.9300	C20—C22	1.538 (5)
C4—C5	1.536 (4)	C20—C21	1.538 (4)
C4—H4B	0.9800	C21—H21A	0.9600
C5—C6	1.463 (4)	C21—H21B	0.9600
C5—C10	1.537 (4)	C21—H21C	0.9600
C6—C7	1.496 (4)	C22—C23	1.511 (4)
C6—H6A	0.9800	C22—H22A	0.9800
C7—C8	1.526 (4)	C23—C24	1.525 (5)
C7—H7A	0.9700	C23—H23A	0.9700
C7—H7B	0.9700	C23—H23B	0.9700
C8—C14	1.533 (3)	C24—C28	1.511 (4)
C8—C9	1.539 (4)	C24—C25	1.537 (4)
C8—H8A	0.9800	C25—C27	1.515 (5)
C9—C11	1.541 (4)	C25—C26	1.554 (4)
C9—C10	1.570 (3)	C27—H27A	0.9600
C9—H9A	0.9800	C27—H27B	0.9600
C10—C19	1.553 (4)	C27—H27C	0.9600
C11—C12	1.535 (4)	C28—H28A	0.9600
C11—H11A	0.9700	C28—H28B	0.9600
C11—H11B	0.9700	C28—H28C	0.9600
C12—C13	1.522 (4)	C1S—Cl1	1.751 (5)
C12—H12A	0.9700	C1S—Cl3	1.753 (6)
C12—H12B	0.9700	C1S—Cl2	1.758 (5)
C13—C18	1.539 (4)	C1S—H1SA	0.9800
C13—C14	1.543 (4)		
			
C26—O2—C22	120.5 (2)	C16—C15—H15B	111.0
C24—O3—C20	110.5 (2)	H15A—C15—H15B	109.0
C4—O4—H4	112 (4)	C15—C16—C17	105.8 (2)
C25—O25—H25	109 (4)	C15—C16—H16A	110.6
C5—O56—C6	60.70 (19)	C17—C16—H16A	110.6
O1—C1—C2	118.9 (3)	C15—C16—H16B	110.6
O1—C1—C10	121.9 (3)	C17—C16—H16B	110.6
C2—C1—C10	119.2 (3)	H16A—C16—H16B	108.7
C3—C2—C1	123.3 (3)	C20—C17—C16	114.3 (2)
C3—C2—H2A	118.3	C20—C17—C13	121.9 (2)
C1—C2—H2A	118.3	C16—C17—C13	103.8 (2)
C2—C3—C4	123.1 (3)	C20—C17—H17A	105.1
C2—C3—H3A	118.5	C16—C17—H17A	105.1
C4—C3—H3A	118.5	C13—C17—H17A	105.1
O4—C4—C3	105.7 (3)	C13—C18—H18A	109.5
O4—C4—C5	111.8 (3)	C13—C18—H18B	109.5
C3—C4—C5	114.3 (3)	H18A—C18—H18B	109.5
O4—C4—H4B	108.3	C13—C18—H18C	109.5
C3—C4—H4B	108.3	H18A—C18—H18C	109.5
C5—C4—H4B	108.3	H18B—C18—H18C	109.5
O56—C5—C6	59.7 (2)	C10—C19—H19A	109.5
O56—C5—C4	108.1 (2)	C10—C19—H19B	109.5
C6—C5—C4	117.8 (3)	H19A—C19—H19B	109.5
O56—C5—C10	116.2 (2)	C10—C19—H19C	109.5
C6—C5—C10	121.1 (2)	H19A—C19—H19C	109.5
C4—C5—C10	118.2 (3)	H19B—C19—H19C	109.5
O56—C6—C5	59.61 (19)	O3—C20—C22	101.0 (2)
O56—C6—C7	113.8 (2)	O3—C20—C21	108.2 (2)
C5—C6—C7	122.6 (2)	C22—C20—C21	110.2 (3)
O56—C6—H6A	116.1	O3—C20—C17	105.5 (2)
C5—C6—H6A	116.1	C22—C20—C17	115.1 (2)
C7—C6—H6A	116.1	C21—C20—C17	115.5 (2)
C6—C7—C8	111.5 (2)	C20—C21—H21A	109.5
C6—C7—H7A	109.3	C20—C21—H21B	109.5
C8—C7—H7A	109.3	H21A—C21—H21B	109.5
C6—C7—H7B	109.3	C20—C21—H21C	109.5
C8—C7—H7B	109.3	H21A—C21—H21C	109.5
H7A—C7—H7B	108.0	H21B—C21—H21C	109.5
C7—C8—C14	109.5 (2)	O2—C22—C23	108.6 (2)
C7—C8—C9	110.8 (2)	O2—C22—C20	110.4 (2)
C14—C8—C9	107.9 (2)	C23—C22—C20	102.6 (3)
C7—C8—H8A	109.5	O2—C22—H22A	111.6
C14—C8—H8A	109.5	C23—C22—H22A	111.6
C9—C8—H8A	109.5	C20—C22—H22A	111.6
C8—C9—C11	111.5 (2)	C22—C23—C24	99.3 (2)
C8—C9—C10	114.9 (2)	C22—C23—H23A	111.9
C11—C9—C10	112.6 (2)	C24—C23—H23A	111.9
C8—C9—H9A	105.6	C22—C23—H23B	111.9
C11—C9—H9A	105.6	C24—C23—H23B	111.9
C10—C9—H9A	105.6	H23A—C23—H23B	109.6
C5—C10—C1	109.0 (2)	O3—C24—C28	109.7 (3)
C5—C10—C19	107.0 (2)	O3—C24—C23	105.2 (2)
C1—C10—C19	106.1 (3)	C28—C24—C23	114.9 (3)
C5—C10—C9	113.1 (2)	O3—C24—C25	104.9 (2)
C1—C10—C9	108.2 (2)	C28—C24—C25	114.1 (3)
C19—C10—C9	113.2 (2)	C23—C24—C25	107.1 (3)
C12—C11—C9	113.0 (2)	O25—C25—C27	111.3 (3)
C12—C11—H11A	109.0	O25—C25—C24	107.0 (2)
C9—C11—H11A	109.0	C27—C25—C24	113.8 (3)
C12—C11—H11B	109.0	O25—C25—C26	106.3 (3)
C9—C11—H11B	109.0	C27—C25—C26	108.6 (3)
H11A—C11—H11B	107.8	C24—C25—C26	109.6 (2)
C13—C12—C11	111.5 (3)	O26—C26—O2	117.3 (3)
C13—C12—H12A	109.3	O26—C26—C25	121.5 (3)
C11—C12—H12A	109.3	O2—C26—C25	121.1 (3)
C13—C12—H12B	109.3	C25—C27—H27A	109.5
C11—C12—H12B	109.3	C25—C27—H27B	109.5
H12A—C12—H12B	108.0	H27A—C27—H27B	109.5
C12—C13—C18	111.1 (3)	C25—C27—H27C	109.5
C12—C13—C14	107.2 (2)	H27A—C27—H27C	109.5
C18—C13—C14	110.7 (2)	H27B—C27—H27C	109.5
C12—C13—C17	116.1 (2)	C24—C28—H28A	109.5
C18—C13—C17	112.8 (2)	C24—C28—H28B	109.5
C14—C13—C17	98.0 (2)	H28A—C28—H28B	109.5
C8—C14—C15	118.9 (2)	C24—C28—H28C	109.5
C8—C14—C13	114.5 (2)	H28A—C28—H28C	109.5
C15—C14—C13	104.6 (2)	H28B—C28—H28C	109.5
C8—C14—H14A	106.0	Cl1—C1S—Cl3	110.3 (3)
C15—C14—H14A	106.0	Cl1—C1S—Cl2	110.3 (3)
C13—C14—H14A	106.0	Cl3—C1S—Cl2	109.7 (3)
C14—C15—C16	104.0 (2)	Cl1—C1S—H1SA	108.8
C14—C15—H15A	111.0	Cl3—C1S—H1SA	108.8
C16—C15—H15A	111.0	Cl2—C1S—H1SA	108.8
C14—C15—H15B	111.0		
			
O1—C1—C2—C3	168.0 (4)	C18—C13—C14—C8	61.1 (3)
C10—C1—C2—C3	−13.6 (6)	C17—C13—C14—C8	179.2 (2)
C1—C2—C3—C4	1.4 (7)	C12—C13—C14—C15	167.9 (2)
C2—C3—C4—O4	113.0 (4)	C18—C13—C14—C15	−70.8 (3)
C2—C3—C4—C5	−10.3 (6)	C17—C13—C14—C15	47.4 (3)
C6—O56—C5—C4	112.2 (3)	C8—C14—C15—C16	−161.5 (3)
C6—O56—C5—C10	−112.2 (3)	C13—C14—C15—C16	−32.3 (3)
O4—C4—C5—O56	46.6 (4)	C14—C15—C16—C17	3.4 (3)
C3—C4—C5—O56	166.5 (3)	C15—C16—C17—C20	161.1 (3)
O4—C4—C5—C6	111.2 (3)	C15—C16—C17—C13	26.1 (3)
C3—C4—C5—C6	−128.8 (3)	C12—C13—C17—C20	71.2 (3)
O4—C4—C5—C10	−88.0 (4)	C18—C13—C17—C20	−58.6 (4)
C3—C4—C5—C10	32.0 (4)	C14—C13—C17—C20	−175.1 (2)
C5—O56—C6—C7	115.1 (3)	C12—C13—C17—C16	−158.1 (3)
C4—C5—C6—O56	−95.6 (3)	C18—C13—C17—C16	72.1 (3)
C10—C5—C6—O56	104.1 (3)	C14—C13—C17—C16	−44.4 (3)
O56—C5—C6—C7	−100.4 (3)	C24—O3—C20—C22	18.6 (3)
C4—C5—C6—C7	164.0 (3)	C24—O3—C20—C21	−97.2 (3)
C10—C5—C6—C7	3.8 (5)	C24—O3—C20—C17	138.7 (2)
O56—C6—C7—C8	−43.7 (3)	C16—C17—C20—O3	56.0 (3)
C5—C6—C7—C8	24.3 (4)	C13—C17—C20—O3	−177.9 (2)
C6—C7—C8—C14	−172.2 (2)	C16—C17—C20—C22	166.4 (2)
C6—C7—C8—C9	−53.3 (3)	C13—C17—C20—C22	−67.5 (3)
C7—C8—C9—C11	−173.0 (2)	C16—C17—C20—C21	−63.3 (4)
C14—C8—C9—C11	−53.1 (3)	C13—C17—C20—C21	62.7 (4)
C7—C8—C9—C10	57.3 (3)	C26—O2—C22—C23	38.6 (4)
C14—C8—C9—C10	177.2 (2)	C26—O2—C22—C20	−73.1 (3)
O56—C5—C10—C1	−172.3 (2)	O3—C20—C22—O2	75.4 (3)
C6—C5—C10—C1	118.8 (3)	C21—C20—C22—O2	−170.3 (2)
C4—C5—C10—C1	−41.4 (4)	C17—C20—C22—O2	−37.6 (3)
O56—C5—C10—C19	−58.1 (3)	O3—C20—C22—C23	−40.2 (3)
C6—C5—C10—C19	−126.9 (3)	C21—C20—C22—C23	74.1 (3)
C4—C5—C10—C19	72.9 (3)	C17—C20—C22—C23	−153.2 (2)
O56—C5—C10—C9	67.3 (3)	O2—C22—C23—C24	−71.4 (3)
C6—C5—C10—C9	−1.6 (4)	C20—C22—C23—C24	45.4 (3)
C4—C5—C10—C9	−161.8 (3)	C20—O3—C24—C28	133.8 (3)
O1—C1—C10—C5	−149.7 (4)	C20—O3—C24—C23	9.7 (3)
C2—C1—C10—C5	32.0 (4)	C20—O3—C24—C25	−103.1 (3)
O1—C1—C10—C19	95.4 (4)	C22—C23—C24—O3	−34.1 (3)
C2—C1—C10—C19	−82.9 (4)	C22—C23—C24—C28	−154.9 (3)
O1—C1—C10—C9	−26.4 (5)	C22—C23—C24—C25	77.2 (3)
C2—C1—C10—C9	155.4 (3)	O3—C24—C25—O25	178.9 (3)
C8—C9—C10—C5	−28.7 (3)	C28—C24—C25—O25	−60.9 (4)
C11—C9—C10—C5	−157.8 (2)	C23—C24—C25—O25	67.4 (3)
C8—C9—C10—C1	−149.5 (2)	O3—C24—C25—C27	−57.7 (3)
C11—C9—C10—C1	81.3 (3)	C28—C24—C25—C27	62.4 (4)
C8—C9—C10—C19	93.2 (3)	C23—C24—C25—C27	−169.3 (3)
C11—C9—C10—C19	−35.9 (3)	O3—C24—C25—C26	64.1 (3)
C8—C9—C11—C12	53.6 (3)	C28—C24—C25—C26	−175.8 (3)
C10—C9—C11—C12	−175.6 (3)	C23—C24—C25—C26	−47.4 (3)
C9—C11—C12—C13	−55.3 (4)	C22—O2—C26—O26	176.1 (3)
C11—C12—C13—C18	−65.2 (3)	C22—O2—C26—C25	−7.0 (4)
C11—C12—C13—C14	55.9 (3)	O25—C25—C26—O26	72.9 (4)
C11—C12—C13—C17	164.2 (3)	C27—C25—C26—O26	−46.8 (4)
C7—C8—C14—C15	−55.9 (3)	C24—C25—C26—O26	−171.7 (3)
C9—C8—C14—C15	−176.6 (3)	O25—C25—C26—O2	−103.9 (3)
C7—C8—C14—C13	179.6 (2)	C27—C25—C26—O2	136.4 (3)
C9—C8—C14—C13	58.9 (3)	C24—C25—C26—O2	11.5 (4)
C12—C13—C14—C8	−60.3 (3)		

### (17S,20R,22R,24R,25S)-5β,6β:20,24-Diepoxy-4β,25-dihydroxy-1-oxowith-2-en-26,22-olide chloroform solvate (I) Hydrogen-bond geometry (Å, º)

**Table:**

*D*—H···*A*	*D*—H	H···*A*	*D*···*A*	*D*—H···*A*
O4—H4···O26^i^	0.79 (6)	2.10 (6)	2.819 (4)	151
O25—H25···O56^ii^	0.74 (5)	2.12 (5)	2.856 (4)	169
C23—H23*A*···O26^iii^	0.97	2.57	3.473 (4)	154
C1*S*—H1*SA*···O26	0.98	2.43	3.393 (6)	168

Symmetry codes: (i) *x*, *y*, *z*+1; (ii) *x*, *y*, *z*−1; (iii) *x*+1, *y*, *z*.

### (20R,22R)-5α,14α,20-Trihydroxy-1-oxo-6α,7α-epoxywitha-2-enolide (II) Crystal data

**Table:**

C_28_H_40_O_7_	*F*(000) = 264
*M**_r_* = 488.60	*D*_x_ = 1.312 Mg m^−^^3^
Triclinic, *P*1	Melting point: 528(3) K
*a* = 6.2374 (12) Å	Cu *K*α radiation, λ = 1.54184 Å
*b* = 9.5938 (19) Å	Cell parameters from 1941 reflections
*c* = 11.351 (2) Å	θ = 4.3–75.5°
α = 112.81 (3)°	µ = 0.76 mm^−^^1^
β = 96.49 (3)°	*T* = 290 K
γ = 93.13 (3)°	Prizmatic, colorless
*V* = 618.5 (2) Å^3^	0.42 × 0.28 × 0.21 mm
*Z* = 1	

### (20R,22R)-5α,14α,20-Trihydroxy-1-oxo-6α,7α-epoxywitha-2-enolide (II) Data collection

**Table:**

Rigaku Xcalibur, Ruby diffractometer	2812 independent reflections
Radiation source: Enhance (Cu) X-ray Source	2545 reflections with *I* > 2σ(*I*)
Graphite monochromator	*R*_int_ = 0.025
Detector resolution: 10.2576 pixels mm^-1^	θ_max_ = 76.7°, θ_min_ = 4.3°
ω scans	*h* = −7→7
Absorption correction: multi-scan (SADABS; Sheldrick, 2003)	*k* = −12→11
*T*_min_ = 0.776, *T*_max_ = 0.853	*l* = −14→10
4169 measured reflections	

### (20R,22R)-5α,14α,20-Trihydroxy-1-oxo-6α,7α-epoxywitha-2-enolide (II) Refinement

**Table:**

Refinement on *F*^2^	Hydrogen site location: mixed
Least-squares matrix: full	H atoms treated by a mixture of independent and constrained refinement
*R*[*F*^2^ > 2σ(*F*^2^)] = 0.041	*w* = 1/[σ^2^(*F*_o_^2^) + (0.0647*P*)^2^ + 0.0383*P*] where *P* = (*F*_o_^2^ + 2*F*_c_^2^)/3
*wR*(*F*^2^) = 0.110	(Δ/σ)_max_ < 0.001
*S* = 1.03	Δρ_max_ = 0.17 e Å^−^^3^
2812 reflections	Δρ_min_ = −0.19 e Å^−^^3^
333 parameters	Absolute structure: Classical Flack method preferred over Parsons because s.u. lower.
3 restraints	Absolute structure parameter: −0.1 (2)
Primary atom site location: structure-invariant direct methods	

### (20R,22R)-5α,14α,20-Trihydroxy-1-oxo-6α,7α-epoxywitha-2-enolide (II) Special details

**Table:**

Geometry. All esds (except the esd in the dihedral angle between two l.s. planes) are estimated using the full covariance matrix. The cell esds are taken into account individually in the estimation of esds in distances, angles and torsion angles; correlations between esds in cell parameters are only used when they are defined by crystal symmetry. An approximate (isotropic) treatment of cell esds is used for estimating esds involving l.s. planes.

### (20R,22R)-5α,14α,20-Trihydroxy-1-oxo-6α,7α-epoxywitha-2-enolide (II) Fractional atomic coordinates and isotropic or equivalent isotropic displacement parameters (Å^2^)

**Table:**

	*x*	*y*	*z*	*U*_iso_*/*U*_eq_	
O1	1.0734 (4)	1.2216 (4)	0.3845 (3)	0.0674 (8)	
O2	1.0070 (4)	0.6040 (2)	0.9120 (2)	0.0441 (5)	
O5	0.8575 (4)	0.8791 (3)	0.0969 (2)	0.0524 (6)	
O14	0.9050 (4)	0.6585 (3)	0.3671 (2)	0.0468 (5)	
O20	0.7306 (4)	0.7378 (3)	0.8118 (2)	0.0444 (5)	
O26	1.0412 (5)	0.5281 (3)	1.0706 (2)	0.0591 (7)	
O67	0.6045 (4)	0.6709 (3)	0.1319 (2)	0.0555 (6)	
C1	0.9157 (5)	1.1689 (4)	0.3017 (3)	0.0431 (7)	
C2	0.8775 (6)	1.2312 (5)	0.2017 (4)	0.0551 (9)	
H2A	0.9624	1.3185	0.2108	0.066*	
C3	0.7256 (6)	1.1664 (5)	0.0993 (4)	0.0605 (10)	
H3A	0.7103	1.2095	0.0387	0.073*	
C4	0.5794 (6)	1.0290 (4)	0.0768 (3)	0.0534 (8)	
H4A	0.5570	0.9639	−0.0146	0.064*	
H4B	0.4396	1.0589	0.1014	0.064*	
C5	0.6713 (5)	0.9395 (4)	0.1537 (3)	0.0417 (7)	
C6	0.5077 (6)	0.8077 (4)	0.1384 (3)	0.0472 (7)	
H6A	0.3725	0.7928	0.0798	0.057*	
C7	0.4970 (5)	0.7531 (4)	0.2412 (3)	0.0444 (7)	
H7A	0.3550	0.7054	0.2427	0.053*	
C8	0.6366 (4)	0.8339 (3)	0.3704 (3)	0.0346 (6)	
H8A	0.5414	0.8946	0.4290	0.042*	
C9	0.8236 (5)	0.9479 (3)	0.3713 (3)	0.0357 (6)	
H9A	0.9315	0.8878	0.3238	0.043*	
C10	0.7426 (5)	1.0460 (3)	0.2988 (3)	0.0358 (6)	
C11	0.9330 (6)	1.0353 (4)	0.5127 (3)	0.0535 (9)	
H11A	1.0577	1.1011	0.5140	0.064*	
H11B	0.8316	1.0994	0.5613	0.064*	
C12	1.0074 (5)	0.9316 (4)	0.5799 (3)	0.0490 (8)	
H12A	1.0653	0.9934	0.6694	0.059*	
H12B	1.1229	0.8769	0.5387	0.059*	
C13	0.8206 (4)	0.8163 (3)	0.5748 (3)	0.0343 (6)	
C14	0.7262 (4)	0.7268 (3)	0.4299 (3)	0.0360 (6)	
C15	0.5738 (5)	0.5985 (4)	0.4314 (3)	0.0471 (7)	
H15A	0.5507	0.5132	0.3481	0.056*	
H15B	0.4347	0.6329	0.4535	0.056*	
C16	0.6930 (5)	0.5546 (4)	0.5361 (3)	0.0451 (7)	
H16A	0.7500	0.4576	0.4966	0.054*	
H16B	0.5941	0.5457	0.5935	0.054*	
C17	0.8822 (4)	0.6823 (3)	0.6131 (3)	0.0353 (6)	
H17A	1.0134	0.6476	0.5756	0.042*	
C18	0.6480 (6)	0.9019 (4)	0.6520 (3)	0.0478 (7)	
H18A	0.5306	0.8303	0.6479	0.072*	
H18B	0.5940	0.9709	0.6161	0.072*	
H18C	0.7113	0.9579	0.7405	0.072*	
C19	0.5529 (5)	1.1315 (4)	0.3550 (3)	0.0459 (7)	
H19A	0.5891	1.1821	0.4472	0.069*	
H19B	0.4257	1.0605	0.3342	0.069*	
H19C	0.5250	1.2052	0.3189	0.069*	
C20	0.9287 (5)	0.7102 (3)	0.7577 (3)	0.0367 (6)	
C21	1.0997 (5)	0.8455 (4)	0.8335 (3)	0.0458 (7)	
H21A	1.0430	0.9375	0.8361	0.069*	
H21B	1.2274	0.8323	0.7921	0.069*	
H21C	1.1360	0.8517	0.9200	0.069*	
C22	1.0006 (5)	0.5651 (3)	0.7731 (3)	0.0378 (6)	
H22A	0.8854	0.4817	0.7265	0.045*	
C23	1.2147 (6)	0.5111 (4)	0.7296 (3)	0.0461 (7)	
H23A	1.3111	0.5980	0.7375	0.055*	
H23B	1.1877	0.4423	0.6392	0.055*	
C24	1.3270 (5)	0.4300 (3)	0.8076 (3)	0.0404 (6)	
H24A	1.4316	0.5062	0.8766	0.048*	
C25	1.1654 (5)	0.3683 (4)	0.8731 (3)	0.0430 (7)	
H25A	1.0508	0.3003	0.8057	0.052*	
C26	1.0632 (5)	0.5015 (4)	0.9602 (3)	0.0414 (7)	
C27	1.2617 (7)	0.2800 (5)	0.9482 (4)	0.0610 (9)	
H27A	1.1503	0.2463	0.9859	0.091*	
H27B	1.3742	0.3445	1.0153	0.091*	
H27C	1.3216	0.1935	0.8909	0.091*	
C28	1.4558 (6)	0.3074 (4)	0.7249 (4)	0.0525 (8)	
H28A	1.5525	0.2760	0.7797	0.079*	
H28B	1.5384	0.3474	0.6764	0.079*	
H28C	1.3576	0.2216	0.6666	0.079*	
H5	0.858 (7)	0.793 (6)	0.113 (4)	0.069 (13)*	
H14	0.884 (8)	0.628 (6)	0.289 (5)	0.069 (14)*	
H20	0.768 (9)	0.767 (7)	0.884 (6)	0.09 (2)*	

### (20R,22R)-5α,14α,20-Trihydroxy-1-oxo-6α,7α-epoxywitha-2-enolide (II) Atomic displacement parameters (Å^2^)

**Table:**

	*U*^11^	*U*^22^	*U*^33^	*U*^12^	*U*^13^	*U*^23^
O1	0.0566 (14)	0.0798 (18)	0.0761 (19)	−0.0157 (13)	−0.0101 (13)	0.0506 (16)
O2	0.0575 (13)	0.0457 (12)	0.0372 (11)	0.0172 (10)	0.0157 (9)	0.0214 (10)
O5	0.0675 (15)	0.0570 (15)	0.0387 (12)	0.0261 (12)	0.0205 (10)	0.0197 (11)
O14	0.0572 (14)	0.0504 (13)	0.0346 (12)	0.0207 (11)	0.0123 (10)	0.0153 (10)
O20	0.0445 (12)	0.0573 (14)	0.0371 (12)	0.0169 (10)	0.0125 (9)	0.0218 (11)
O26	0.0718 (17)	0.0731 (17)	0.0456 (14)	0.0210 (13)	0.0187 (12)	0.0335 (13)
O67	0.0801 (17)	0.0401 (12)	0.0352 (12)	0.0108 (11)	−0.0037 (11)	0.0057 (9)
C1	0.0428 (16)	0.0457 (17)	0.0456 (17)	0.0093 (13)	0.0078 (13)	0.0222 (14)
C2	0.059 (2)	0.058 (2)	0.063 (2)	0.0067 (16)	0.0102 (17)	0.0395 (19)
C3	0.070 (2)	0.071 (2)	0.059 (2)	0.0197 (19)	0.0074 (18)	0.044 (2)
C4	0.065 (2)	0.059 (2)	0.0408 (17)	0.0164 (17)	−0.0008 (15)	0.0253 (16)
C5	0.0501 (17)	0.0462 (17)	0.0301 (14)	0.0166 (14)	0.0064 (12)	0.0148 (13)
C6	0.0545 (18)	0.0424 (17)	0.0366 (15)	0.0054 (13)	−0.0093 (13)	0.0112 (13)
C7	0.0449 (16)	0.0414 (17)	0.0397 (16)	0.0014 (13)	−0.0063 (13)	0.0121 (13)
C8	0.0354 (14)	0.0361 (14)	0.0293 (14)	0.0030 (11)	0.0002 (11)	0.0110 (11)
C9	0.0377 (14)	0.0393 (15)	0.0300 (13)	0.0042 (11)	0.0031 (10)	0.0142 (12)
C10	0.0387 (14)	0.0382 (14)	0.0315 (13)	0.0074 (11)	0.0057 (10)	0.0145 (11)
C11	0.070 (2)	0.0465 (18)	0.0395 (17)	−0.0196 (16)	−0.0160 (16)	0.0223 (15)
C12	0.0541 (19)	0.0528 (19)	0.0384 (16)	−0.0158 (15)	−0.0106 (13)	0.0238 (15)
C13	0.0405 (15)	0.0327 (13)	0.0299 (13)	0.0017 (11)	0.0014 (11)	0.0141 (11)
C14	0.0381 (14)	0.0351 (14)	0.0305 (14)	0.0032 (11)	0.0030 (11)	0.0090 (11)
C15	0.0500 (18)	0.0387 (15)	0.0466 (17)	−0.0078 (13)	−0.0078 (13)	0.0161 (14)
C16	0.0537 (18)	0.0377 (16)	0.0410 (16)	−0.0037 (13)	0.0007 (13)	0.0153 (13)
C17	0.0382 (14)	0.0354 (14)	0.0321 (14)	0.0030 (11)	0.0067 (11)	0.0128 (12)
C18	0.065 (2)	0.0458 (17)	0.0371 (16)	0.0202 (15)	0.0126 (14)	0.0179 (14)
C19	0.0497 (18)	0.0423 (16)	0.0476 (18)	0.0135 (14)	0.0146 (14)	0.0167 (14)
C20	0.0390 (14)	0.0392 (14)	0.0313 (14)	0.0046 (11)	0.0066 (11)	0.0130 (12)
C21	0.0556 (19)	0.0444 (16)	0.0356 (15)	−0.0026 (14)	−0.0020 (13)	0.0171 (13)
C22	0.0416 (15)	0.0408 (15)	0.0311 (14)	0.0043 (12)	0.0054 (11)	0.0145 (12)
C23	0.0491 (17)	0.0510 (18)	0.0441 (17)	0.0133 (14)	0.0138 (13)	0.0223 (15)
C24	0.0429 (15)	0.0365 (14)	0.0404 (15)	0.0057 (12)	0.0044 (12)	0.0141 (12)
C25	0.0443 (15)	0.0414 (16)	0.0460 (17)	0.0057 (12)	0.0047 (13)	0.0207 (14)
C26	0.0421 (15)	0.0464 (17)	0.0419 (16)	0.0052 (13)	0.0091 (12)	0.0235 (14)
C27	0.075 (2)	0.055 (2)	0.068 (2)	0.0181 (19)	0.0158 (19)	0.0374 (19)
C28	0.0481 (17)	0.0475 (18)	0.063 (2)	0.0124 (14)	0.0125 (15)	0.0211 (16)

### (20R,22R)-5α,14α,20-Trihydroxy-1-oxo-6α,7α-epoxywitha-2-enolide (II) Geometric parameters (Å, º)

**Table:**

O1—C1	1.214 (4)	C13—C18	1.534 (4)
O2—C26	1.341 (4)	C13—C14	1.555 (4)
O2—C22	1.468 (3)	C13—C17	1.562 (4)
O5—C5	1.433 (4)	C14—C15	1.520 (4)
O5—H5	0.92 (5)	C15—C16	1.538 (5)
O14—C14	1.442 (4)	C15—H15A	0.9700
O14—H14	0.81 (5)	C15—H15B	0.9700
O20—C20	1.434 (4)	C16—C17	1.561 (4)
O20—H20	0.76 (6)	C16—H16A	0.9700
O26—C26	1.204 (4)	C16—H16B	0.9700
O67—C7	1.448 (4)	C17—C20	1.546 (4)
O67—C6	1.454 (4)	C17—H17A	0.9800
C1—C2	1.478 (5)	C18—H18A	0.9600
C1—C10	1.542 (4)	C18—H18B	0.9600
C2—C3	1.327 (6)	C18—H18C	0.9600
C2—H2A	0.9300	C19—H19A	0.9600
C3—C4	1.480 (6)	C19—H19B	0.9600
C3—H3A	0.9300	C19—H19C	0.9600
C4—C5	1.532 (4)	C20—C21	1.530 (4)
C4—H4A	0.9700	C20—C22	1.552 (4)
C4—H4B	0.9700	C21—H21A	0.9600
C5—C6	1.523 (5)	C21—H21B	0.9600
C5—C10	1.560 (4)	C21—H21C	0.9600
C6—C7	1.458 (5)	C22—C23	1.523 (4)
C6—H6A	0.9800	C22—H22A	0.9800
C7—C8	1.506 (4)	C23—C24	1.527 (5)
C7—H7A	0.9800	C23—H23A	0.9700
C8—C14	1.530 (4)	C23—H23B	0.9700
C8—C9	1.551 (4)	C24—C28	1.526 (5)
C8—H8A	0.9800	C24—C25	1.539 (4)
C9—C10	1.542 (4)	C24—H24A	0.9800
C9—C11	1.546 (4)	C25—C26	1.507 (5)
C9—H9A	0.9800	C25—C27	1.518 (5)
C10—C19	1.534 (4)	C25—H25A	0.9800
C11—C12	1.532 (5)	C27—H27A	0.9600
C11—H11A	0.9700	C27—H27B	0.9600
C11—H11B	0.9700	C27—H27C	0.9600
C12—C13	1.542 (4)	C28—H28A	0.9600
C12—H12A	0.9700	C28—H28B	0.9600
C12—H12B	0.9700	C28—H28C	0.9600
			
C26—O2—C22	119.2 (2)	C14—C15—H15A	111.0
C5—O5—H5	100 (3)	C16—C15—H15A	111.0
C14—O14—H14	114 (3)	C14—C15—H15B	111.0
C20—O20—H20	103 (4)	C16—C15—H15B	111.0
C7—O67—C6	60.3 (2)	H15A—C15—H15B	109.0
O1—C1—C2	118.9 (3)	C15—C16—C17	107.5 (2)
O1—C1—C10	123.8 (3)	C15—C16—H16A	110.2
C2—C1—C10	117.1 (3)	C17—C16—H16A	110.2
C3—C2—C1	122.0 (3)	C15—C16—H16B	110.2
C3—C2—H2A	119.0	C17—C16—H16B	110.2
C1—C2—H2A	119.0	H16A—C16—H16B	108.5
C2—C3—C4	123.3 (3)	C20—C17—C16	113.2 (2)
C2—C3—H3A	118.3	C20—C17—C13	119.4 (2)
C4—C3—H3A	118.3	C16—C17—C13	103.1 (2)
C3—C4—C5	112.1 (3)	C20—C17—H17A	106.8
C3—C4—H4A	109.2	C16—C17—H17A	106.8
C5—C4—H4A	109.2	C13—C17—H17A	106.8
C3—C4—H4B	109.2	C13—C18—H18A	109.5
C5—C4—H4B	109.2	C13—C18—H18B	109.5
H4A—C4—H4B	107.9	H18A—C18—H18B	109.5
O5—C5—C6	108.7 (3)	C13—C18—H18C	109.5
O5—C5—C4	105.4 (3)	H18A—C18—H18C	109.5
C6—C5—C4	110.6 (3)	H18B—C18—H18C	109.5
O5—C5—C10	109.5 (2)	C10—C19—H19A	109.5
C6—C5—C10	111.4 (2)	C10—C19—H19B	109.5
C4—C5—C10	111.0 (3)	H19A—C19—H19B	109.5
O67—C6—C7	59.6 (2)	C10—C19—H19C	109.5
O67—C6—C5	114.1 (3)	H19A—C19—H19C	109.5
C7—C6—C5	121.6 (3)	H19B—C19—H19C	109.5
O67—C6—H6A	116.3	O20—C20—C21	109.3 (3)
C7—C6—H6A	116.3	O20—C20—C17	108.7 (2)
C5—C6—H6A	116.3	C21—C20—C17	112.1 (2)
O67—C7—C6	60.0 (2)	O20—C20—C22	105.9 (2)
O67—C7—C8	117.5 (3)	C21—C20—C22	110.1 (2)
C6—C7—C8	120.6 (3)	C17—C20—C22	110.5 (2)
O67—C7—H7A	115.7	C20—C21—H21A	109.5
C6—C7—H7A	115.7	C20—C21—H21B	109.5
C8—C7—H7A	115.7	H21A—C21—H21B	109.5
C7—C8—C14	113.8 (2)	C20—C21—H21C	109.5
C7—C8—C9	114.2 (2)	H21A—C21—H21C	109.5
C14—C8—C9	109.8 (2)	H21B—C21—H21C	109.5
C7—C8—H8A	106.1	O2—C22—C23	110.7 (2)
C14—C8—H8A	106.1	O2—C22—C20	103.0 (2)
C9—C8—H8A	106.1	C23—C22—C20	118.2 (2)
C10—C9—C11	116.2 (2)	O2—C22—H22A	108.2
C10—C9—C8	111.0 (2)	C23—C22—H22A	108.2
C11—C9—C8	108.0 (2)	C20—C22—H22A	108.2
C10—C9—H9A	107.1	C22—C23—C24	113.1 (3)
C11—C9—H9A	107.1	C22—C23—H23A	109.0
C8—C9—H9A	107.1	C24—C23—H23A	109.0
C19—C10—C9	111.9 (2)	C22—C23—H23B	109.0
C19—C10—C1	105.6 (2)	C24—C23—H23B	109.0
C9—C10—C1	114.3 (2)	H23A—C23—H23B	107.8
C19—C10—C5	110.1 (2)	C28—C24—C23	111.1 (3)
C9—C10—C5	108.3 (2)	C28—C24—C25	112.6 (3)
C1—C10—C5	106.5 (2)	C23—C24—C25	111.5 (3)
C12—C11—C9	113.7 (3)	C28—C24—H24A	107.1
C12—C11—H11A	108.8	C23—C24—H24A	107.1
C9—C11—H11A	108.8	C25—C24—H24A	107.1
C12—C11—H11B	108.8	C26—C25—C27	110.4 (3)
C9—C11—H11B	108.8	C26—C25—C24	107.4 (2)
H11A—C11—H11B	107.7	C27—C25—C24	115.1 (3)
C11—C12—C13	112.1 (3)	C26—C25—H25A	107.9
C11—C12—H12A	109.2	C27—C25—H25A	107.9
C13—C12—H12A	109.2	C24—C25—H25A	107.9
C11—C12—H12B	109.2	O26—C26—O2	118.0 (3)
C13—C12—H12B	109.2	O26—C26—C25	125.7 (3)
H12A—C12—H12B	107.9	O2—C26—C25	116.2 (3)
C18—C13—C12	109.4 (3)	C25—C27—H27A	109.5
C18—C13—C14	111.2 (2)	C25—C27—H27B	109.5
C12—C13—C14	106.9 (2)	H27A—C27—H27B	109.5
C18—C13—C17	111.2 (2)	C25—C27—H27C	109.5
C12—C13—C17	117.4 (2)	H27A—C27—H27C	109.5
C14—C13—C17	100.3 (2)	H27B—C27—H27C	109.5
O14—C14—C15	107.0 (2)	C24—C28—H28A	109.5
O14—C14—C8	109.7 (2)	C24—C28—H28B	109.5
C15—C14—C8	119.1 (2)	H28A—C28—H28B	109.5
O14—C14—C13	105.9 (2)	C24—C28—H28C	109.5
C15—C14—C13	103.5 (2)	H28A—C28—H28C	109.5
C8—C14—C13	110.8 (2)	H28B—C28—H28C	109.5
C14—C15—C16	103.8 (2)		
			
O1—C1—C2—C3	171.0 (4)	C9—C8—C14—O14	−52.5 (3)
C10—C1—C2—C3	−12.6 (5)	C7—C8—C14—C15	−46.7 (4)
C1—C2—C3—C4	1.1 (6)	C9—C8—C14—C15	−176.2 (3)
C2—C3—C4—C5	−20.5 (6)	C7—C8—C14—C13	−166.4 (3)
C3—C4—C5—O5	−68.1 (4)	C9—C8—C14—C13	64.1 (3)
C3—C4—C5—C6	174.6 (3)	C18—C13—C14—O14	177.1 (2)
C3—C4—C5—C10	50.3 (4)	C12—C13—C14—O14	57.8 (3)
C7—O67—C6—C5	113.8 (3)	C17—C13—C14—O14	−65.2 (3)
O5—C5—C6—O67	27.8 (3)	C18—C13—C14—C15	−70.5 (3)
C4—C5—C6—O67	143.1 (3)	C12—C13—C14—C15	170.1 (3)
C10—C5—C6—O67	−92.9 (3)	C17—C13—C14—C15	47.2 (3)
O5—C5—C6—C7	95.8 (4)	C18—C13—C14—C8	58.2 (3)
C4—C5—C6—C7	−148.9 (3)	C12—C13—C14—C8	−61.2 (3)
C10—C5—C6—C7	−25.0 (4)	C17—C13—C14—C8	175.9 (2)
C6—O67—C7—C8	−111.3 (3)	O14—C14—C15—C16	74.2 (3)
C5—C6—C7—O67	−101.2 (3)	C8—C14—C15—C16	−160.8 (3)
O67—C6—C7—C8	106.1 (3)	C13—C14—C15—C16	−37.3 (3)
C5—C6—C7—C8	4.9 (5)	C14—C15—C16—C17	13.0 (3)
O67—C7—C8—C14	−71.8 (3)	C15—C16—C17—C20	146.3 (3)
C6—C7—C8—C14	−141.6 (3)	C15—C16—C17—C13	16.0 (3)
O67—C7—C8—C9	55.4 (4)	C18—C13—C17—C20	−46.5 (3)
C6—C7—C8—C9	−14.4 (4)	C12—C13—C17—C20	80.5 (3)
C7—C8—C9—C10	44.7 (3)	C14—C13—C17—C20	−164.2 (2)
C14—C8—C9—C10	173.9 (2)	C18—C13—C17—C16	79.9 (3)
C7—C8—C9—C11	173.1 (3)	C12—C13—C17—C16	−153.1 (3)
C14—C8—C9—C11	−57.7 (3)	C14—C13—C17—C16	−37.8 (3)
C11—C9—C10—C19	−67.2 (3)	C16—C17—C20—O20	−53.7 (3)
C8—C9—C10—C19	56.7 (3)	C13—C17—C20—O20	67.9 (3)
C11—C9—C10—C1	52.9 (4)	C16—C17—C20—C21	−174.6 (3)
C8—C9—C10—C1	176.7 (2)	C13—C17—C20—C21	−53.1 (3)
C11—C9—C10—C5	171.4 (3)	C16—C17—C20—C22	62.1 (3)
C8—C9—C10—C5	−64.8 (3)	C13—C17—C20—C22	−176.3 (2)
O1—C1—C10—C19	99.7 (4)	C26—O2—C22—C23	−53.1 (3)
C2—C1—C10—C19	−76.5 (4)	C26—O2—C22—C20	179.6 (2)
O1—C1—C10—C9	−23.8 (5)	O20—C20—C22—O2	−54.7 (3)
C2—C1—C10—C9	160.0 (3)	C21—C20—C22—O2	63.4 (3)
O1—C1—C10—C5	−143.3 (4)	C17—C20—C22—O2	−172.2 (2)
C2—C1—C10—C5	40.5 (4)	O20—C20—C22—C23	−177.1 (3)
O5—C5—C10—C19	170.9 (3)	C21—C20—C22—C23	−59.1 (3)
C6—C5—C10—C19	−68.9 (3)	C17—C20—C22—C23	65.4 (3)
C4—C5—C10—C19	54.9 (3)	O2—C22—C23—C24	31.1 (4)
O5—C5—C10—C9	−66.6 (3)	C20—C22—C23—C24	149.5 (3)
C6—C5—C10—C9	53.7 (3)	C22—C23—C24—C28	148.4 (3)
C4—C5—C10—C9	177.5 (3)	C22—C23—C24—C25	21.9 (4)
O5—C5—C10—C1	56.8 (3)	C28—C24—C25—C26	175.2 (3)
C6—C5—C10—C1	177.1 (3)	C23—C24—C25—C26	−59.1 (3)
C4—C5—C10—C1	−59.1 (3)	C28—C24—C25—C27	51.8 (4)
C10—C9—C11—C12	179.3 (3)	C23—C24—C25—C27	177.5 (3)
C8—C9—C11—C12	53.9 (4)	C22—O2—C26—O26	−170.9 (3)
C9—C11—C12—C13	−55.1 (4)	C22—O2—C26—C25	13.8 (4)
C11—C12—C13—C18	−64.7 (3)	C27—C25—C26—O26	−6.5 (5)
C11—C12—C13—C14	55.8 (3)	C24—C25—C26—O26	−132.7 (3)
C11—C12—C13—C17	167.4 (3)	C27—C25—C26—O2	168.4 (3)
C7—C8—C14—O14	76.9 (3)	C24—C25—C26—O2	42.2 (4)

### (20R,22R)-5α,14α,20-Trihydroxy-1-oxo-6α,7α-epoxywitha-2-enolide (II) Hydrogen-bond geometry (Å, º)

**Table:**

*D*—H···*A*	*D*—H	H···*A*	*D*···*A*	*D*—H···*A*
O20—H20···O5^i^	0.76 (6)	2.22 (6)	2.973 (4)	173
C7—H7*A*···O26^ii^	0.98	2.59	3.367 (5)	136

Symmetry codes: (i) *x*, *y*, *z*+1; (ii) *x*−1, *y*, *z*−1.
